# Do people become more interested in a healthier lifestyle at temporal landmarks? A comparison between individualistic and collectivistic cultures

**DOI:** 10.3389/fpsyg.2026.1808309

**Published:** 2026-06-22

**Authors:** Bora Moon, Hyunwoong Pyun, Wonseok Jang

**Affiliations:** Department of Sports Science, Sungkyunkwan University, Suwon, Republic of Korea

**Keywords:** collectivism, exercise, fresh start, health, individualism, temporal landmark

## Abstract

**Objectives:**

The objective of this study is to understand whether individuals show different search patterns for health-related terms on Google Trends depending on their cultural background.

**Method:**

This study analyzed Google Trends data for health-related terms in both the United States and South Korea.

**Results:**

The result suggests that Americans show stronger temporal landmark effects than Korean with health-related terms being more heavily searched during earlier time periods. Meanwhile, Koreans also showed an inverted U-shape pattern, where health-related terms were more often searched in the middle of the year than in the early periods.

**Discussion:**

The results offer key implications for policymakers or marketers who are interested in health-focused campaigns, as well as extend the concept of temporal landmarks by comparing the United States and South Korea.

## 1 Introduction

People often set goals as motivational triggers to influence their behaviors (Blythe et al., 2025; Wilson and Brookfield, 2009). Particularly, goals can be set in various ways, such as setting them collectively to motivate one another or by breaking them into subgoals to make progress step-by-step (Burke et al., 2010; Huang et al., 2017). For example, people often join running communities and set daily goals with other members, or create 4-week workout plans with specific sets and reps to achieve their exercise goals. Another distinctive aspect of goal setting is timing (Karniol and Ross, 1996). It is easy to find a person who purchases a gym membership at the early stages of a new period, such as January 1st, to lose weight and adopt a healthier lifestyle. This phenomenon is known as a new year's resolution (i.e., the fresh start phenomenon) (Dai et al., 2014; Yu et al., 2023).

Although goals perform as powerful motivational triggers for health-related activities form a psychological perspective (Jeong et al., 2023), certain aspects have received limited scholarly attention, particularly the temporal dimension. A few notable exceptions include construal level theory which shows how different time frames persuade individuals to construe goals differently and ultimately determine their decision-making (Trope and Liberman, 2010). Furthermore, the concept of temporal landmarks in goal setting has received some scholarly attention which focuses on the role of specific time periods in initiating goals (i.e., the fresh start phenomenon) (Beshears et al., 2021; Yu et al., 2023). In our study, we concentrate on the notion of temporal landmarks, as this framework emphasizes the temporal aspect of goal setting and highlights the crucial role of time selection in establishing goals (Dai et al., 2014), whereas other theories and frameworks treat the temporal dimension merely as a sub-dimension of goal setting (e.g., construal level theory; Trope and Liberman, 2010). In this line of research, studies have examined how the emphasis on temporal landmarks, such as special public events (e.g., national holidays), the starting point of the calendar (e.g., Monday or the 1st day of the month), and meaningful personal events, determines individuals' interest in certain actions and various types of decision making (e.g., financial saving and healthier lifestyles; Bi et al., 2021; Dai et al., 2014; Min et al., 2024). The existing knowledge collectively suggests that the notion of a temporal landmark not only results in more virtuous actions by individuals but also that several mechanisms exist to drive such effects, such as the sufficiency of self-regulatory resources (Yu et al., 2023), the fresh start effect (Dai et al., 2014), and a focus on the big picture (Hennecke and Converse, 2017). In particular, the most powerful mechanism is the fresh start mindset (FSM), which people often experience at the start of a mental cycle from a temporal standpoint (Guo et al., 2025). People develop interest and enhance their motivation to adopt health-related activities when a certain time point on the calendar is mentally construed as a new starting point (Dai et al., 2014).

While the fresh start phenomenon has been demonstrated in multiple studies, a key empirical gap exists from a cultural perspective. Most of the existing knowledge is limited to countries where individualism is prevalent, such as the United States (Beshears et al., 2021; Dai et al., 2014). This limitation leads to interesting research questions about whether the fresh start phenomenon also occurs in collectivistic societies (e.g., South Korea and Japan). In collectivistic societies, interdependent aspects, such as social relationships and social hierarchies, have a stronger effect on individuals' attitudes and behaviors than independent aspects, such as personal goals (Han and Shavitt, 1994). Thus, the FSM, which is activated by personal interests and goals, may be less likely to be evoked in individuals who grew up in collectivistic societies. Due to this cultural difference, comparing the patterns of the fresh start phenomenon across individualistic and collectivistic contexts could offer unique theoretical implications and valuable marketing insights, as the timing of effective marketing campaigns may differ across cultural contexts. In particular, the present research utilizes the United States as a representative example of an individualistic society while using South Korea as a representative example of a collectivistic society. We made this decision based on two different perspectives. First, scholars have selected the United States as a representative individualistic culture and South Korea as a representative collectivistic culture (Kim et al., 2009). Second, the most well-known culture framework, Hofstede's cultural dimensions, has shown that the United States and South Korea have opposite cultural scores across numerous dimensions (e.g., individualism and indulgence; Hofstede Insights, n.d.). This evidence supports our rationale for comparing the search patterns of these two countries.

The present study concentrates on the context of health to examine whether American and Korean show distinctive information search patterns on Google Trends regarding health-related terms including dieting and exercise. In this study, similar to existing literature (e.g., Koo et al., 2020), search patterns are defined as personal interest and curiosity toward health-related activities, rather than behavioral intentions or actual behaviors. This clarification is important, as individuals' interest and motivation, and even behavioral intention do not always lead to actual behavior (Webb and Sheeran, 2006). In a similar vein, existing literature has adopted a similar methodological approach, as Google is the most popular search engine where individuals search for information regarding various activities, particularly those related to health (Choi and Varian, 2012; Dai et al., 2014). Thus, this study examines Google users' search patterns for health-related activities across different time points to determine whether the fresh start phenomenon occurs in both individualistic and collectivistic societies.

## 2 Theoretical background

### 2.1 Goals and temporal landmark

People often commit to goals that provide long-term health benefits (Strecher et al., 1995). In general, individuals' interests in pursuing actions can be determined based on two distinctive systems, such as conscious and unconscious processes (Custers and Aarts, 2010). Studies have demonstrated that individuals with goals show stronger motivation to exercise, particularly when these goals are intrinsically driven (Ryan and Deci, 2000; Wilson and Brookfield, 2009). Given these positive outcomes, scholars have explored numerous ways to enhance motivation to exercise through various strategies, such as setting subgoals, implementing appropriate goal specificity and difficulty, and providing feedback (Burke et al., 2010; Huang et al., 2017). In particular, one key strategy that has received a substantial amount of attention in the health context is the notion of temporal landmarks (Yu et al., 2023). The notion of a temporal landmark is defined as a specific time selection point that creates a mental disconnection among the past, the current, and the future (Dai et al., 2014). In the existing literature, temporal landmarks are conceptualized in two distinct ways (Shum, 1998). Research has demonstrated that emphasizing new time points on the calendar or highlighting meaningful events triggers a FSM (Koo et al., 2020). The former conceptualization focuses on the idea of reference points based on calendar timetables, such as the early stages of the week or year and after a major holiday (Dai et al., 2014). The latter conceptualization emphasizes meaningful life events, such as after a birthday or graduation (Koo et al., 2020). In general, the activation of the FSM performs as a strong motivational source for individuals' interest in health-related activities, such as healthy eating (e.g., consuming virtue foods, Yu et al., 2023), as well as dieting and exercise (Dai et al., 2014). For example, Dai et al. (2014) showed that individuals possess a stronger tendency to initiate their personal goals (e.g., adopting healthier lifestyles) when a FSM is activated. Similarly, Yu et al. (2023) demonstrated that the activation of a FSM motivates individuals to consume virtue foods, which are healthy but less tasty, rather than vice foods, which are tasty but less healthy.

As the power of temporal landmarks has been continuously demonstrated, scholars have become increasingly interested in identifying the key underlying mechanisms of the fresh start phenomenon (Dai et al., 2014). In particular, research has found that the fresh start phenomenon is driven by various processes, such as the mental separation between the past and future (Yu et al., 2023), self-regulatory resources (Kouchaki and Smith, 2014), and attention mechanisms (Soster et al., 2010). Regarding the mental separation mechanism, although individuals generally perceive their past, present, and future as a continuous timeline, the FSM encourages them to view each moment separately and motivates them to compare different time periods (Yu et al., 2023). Consequently, the emphasis on a new beginning enhances their motivation for aspirational behaviors (Dai et al., 2014). For instance, Dai et al. (2015) demonstrated that a FSM activated at the early stages of temporal cycles enhances individuals' intentions to receive goal reminders and increases their motivation to pursue goals compared to situations in which the FSM is inactive. Attention mechanisms also elicit FSM, as temporal landmarks motivate individuals to focus with greater attention on the big picture of their goals, such as processing information from a top-down perspective and at a higher level, while preventing them from focusing on bottom-up and concrete details of goal achievement (Hennecke and Converse, 2017). As a result, this top-down and high level information processing motivates individuals to value the desirability of the goal more highly, which serves as an energy source for motivation rather than the effort (e.g., time and energy) required to achieve it (Beshears et al., 2021). Another mechanism that drives the temporal landmark effect is self-regulatory resources (Kouchaki and Smith, 2014). Research has found that individuals possess greater amounts of energy (i.e., self-regulatory resources) in the early stages of the week than in later periods, which ultimately leads to a preference for virtuous actions (Yu et al., 2023). For instance, Kouchaki and Smith (2014) found that in the early morning, individuals are less likely to commit unethical actions (e.g., lying and cheating) than later time periods, and the reduction of self-regulatory resources and moral awareness contribute to this morning morality effect.

In sum, evidence has continuously demonstrated that the fresh start phenomenon is prevalent in our society and can be activated by numerous mechanisms (Dai et al., 2014; Yu et al., 2023). Nevertheless, there are interesting theoretical questions that remain to be answered in the existing literature. Particularly, it is not clear whether fresh start effects occur across different societies that have distinct cultural mindsets. Investigating the existence of the fresh start phenomenon in both individualistic and collectivistic societies would extend the existing literature as previous studies have mostly tested these effects in individualistic societies (i.e., the United States) (Beshears et al., 2021; Dai et al., 2014).

### 2.2 Cultural values and temporal landmarks

In general, cultural values are categorized into two perspectives: (1) individualism vs. (2) collectivism (Han and Shavitt, 1994). Cross-cultural scholars have asserted that the social structures (e.g., media, family relationships, schools, and workplaces) embedded in each society create these two types of cultural values (Mamo et al., 2023; Oyserman and Lee, 2008; Tsai and Men, 2017). Consequently, these cultural values shape how members of society think, make decisions, and behave (Lee et al., 2018; Odag et al., 2018; Um and Lee, 2015). Individualism is defined by scholars as a cultural value in which people view themselves as unique individuals who possess personal autonomy within social groups (Muralidharan et al., 2017; Triandis, 1989). Scholars have asserted that individualism fosters an independent mindset which leads members of society to emphasize personal goals and individual growth (Oyserman et al., 2002; Soler-Anguiano et al., 2023). In this regard, individuals' own values and beliefs become more important than group norms and collective interests when people in individualistic societies form attitudes and make decisions (Han and Shavitt, 1994). Based on this notion, cross-cultural scholars have found that individuals who grow up in individualistic cultures tend to make decontextualized decisions by prioritizing personal goals and preferences over context specific reasoning (Dutta-Bergman and Wells, 2002). Furthermore, individualists experience greater positive feelings, wellbeing, and life satisfaction when they achieve personal goals and feel positive about themselves (Oyserman et al., 2002). Meanwhile, collectivism is defined as a cultural value in which individuals see themselves as key members of society where members are highly interconnected (Triandis, 1989). In collectivistic societies, people generally adopt an interdependent mindset that guides them to focus more on their relationships with other members of society than on their own personal goals and values (Imada, 2012; Wu et al., 2020). Moreover, social norms and structures, and mutual rules are more valued in collectivistic societies, thus group membership strongly affects individuals' attitudes and identities (Oyserman et al., 2002). Consequently, experiencing group harmony and fulfilling social roles are key components of wellbeing and life satisfaction for individuals who live in collectivistic societies (Ford et al., 2015).

Based on cross-cultural theory, scholars have investigated how individuals from different societies form attitudes and make decisions in distinct ways (Oyserman and Lee, 2008). The fundamental assumption of this line of research is that the cultural components of each society, such as individualism and collectivism, shape individuals' values and goals, which in turn influence their decisions and behaviors (Czarnecka and Schivinski, 2022). For example, Tsai and Men (2017) found that social media users from individualistic and collectivistic cultures use SNSs for different purposes, whereby Chinese users (i.e., from a collectivistic culture) who place greater emphasis on social relationships are more likely to leave comments, reply to questions, share more information on their personal accounts, and form a stronger identification with brands' SNS pages than American users (i.e., from an individualistic culture). Similarly, Jiang et al. (2022) found that the COVID 19 spread in a faster way in individualistic countries, especially in the early stages, than in collectivistic countries because individualists behaved in their own way to value their freedom while collectivists cared more about other society members.

As discussed above, the fresh start phenomenon may occur differently between individualistic and collectivistic cultures, as each society values distinct perspectives (Han and Shavitt, 1994). Thus, we aim to test whether the fresh start phenomenon also occurs within collectivistic societies.

### 2.3 Hypothesis and research question

As discussed earlier, most temporal landmark studies have been conducted in individualistic societies (e.g., the United States), where society emphasizes the importance of personal values and growth (Beshears et al., 2021). In such cultures, people naturally develop an approach-oriented mindset because the future is prioritized more heavily than the past (Guo et al., 2012). Thus, achieving future goals predominantly influences individuals' lives in individualistic societies (Triandis, 1989). In this regard, consistent with the general pattern of the fresh start phenomenon (e.g., Dai et al., 2014), we predict that online search patterns for health-related terms will peak at the initiation of the time period among individuals who live in individualistic cultures. Meanwhile, this study predicts that the fresh start phenomenon may not always occur among individuals who live in collectivistic cultures. Individuals from these societies naturally develop a past-oriented mindset (Guo et al., 2012); thus, past accomplishments are more strongly valued in collectivistic societies than are future goals (Yeganeh, 2024). Furthermore, cultural scholars have found that collectivism is strongly related to interdependent mindsets, which leads individuals to place less importance on time compared to those with independent mindsets (Chan and Saqib, 2022). Moreover, individuals' attitudes and behaviors in collectivistic cultures may be more strongly influenced by the existence of other society members than those in individualistic cultures, as they tend to experience a greater level of social anxiety (Heinrichs et al., 2006). Thus, this study predicts that the fresh start phenomenon is less likely to occur among Koreans who live in a collectivistic culture. Accordingly, we propose Hypothesis 1 based on existing temporal landmark studies and further develop a research question to examine whether the patterns differ between individualistic and collectivistic societies, as follows:

H1: In the United States, people search for “diet” and “exercise” more frequently on Google at the (a) beginning of the week, (b) beginning of the month, and (c) beginning of the year than at the end of the week, end of the month, and end of the year.

RQ: Do the search patterns for “diet” and “exercise” in South Korea align with the fresh start phenomenon, or do they exhibit a distinctive pattern?

## 3 Methods

### 3.1 Study design

To test the hypothesis and research question, this study used web-based search pattern analysis, which is an effective tool for examining individuals' search patterns for health-related terms on Google Trends (Dai et al., 2014). Consistent with our approach, existing research has heavily utilized Google Trends, which shows the relative frequency of target terms across different time periods and regions (Koo et al., 2020), to examine individuals' interests in health-related terms online (Choi and Varian, 2012; Malhotra and Kempegowda, 2023; Nuti et al., 2014). Furthermore, as aforementioned, the current study identifies the United States as a key representative of individualism, while South Korea is selected as a representative of collectivism.

### 3.2 Data collection

Following recommendations from the existing literature (Dai et al., 2014; Koo et al., 2020), this study focuses on “diet” and “exercise” as key terms for health-related activities. Both terms not only represent users' interests in health-related activities but narrowing the focus to these terms may also enhance the internal validity of the findings by controlling for situational noise. Furthermore, this study includes several terms (i.e., news, weather, and YouTube) unrelated to health-related activities as a placebo test to truly demonstrate the fresh start effect. The selection of these placebo terms followed decisions made in past literature (Dai et al., 2014; Koo et al., 2020) and the most common search terms on Google in both the United States and South Korea (Google, n.d.).

In particular, Google Trends provides search pattern data starting from January 1, 2004, using a relative search popularity scale ranging from 0 to 100 (Dai et al., 2014). Specifically, Google Trends values reflect a relative search frequency compared to other searches rather than the absolute search frequency (Jun et al., 2018). In this study, we chose to analyze data beginning in January 2011 rather than January 2004 for the following reasons. The usage rate of Google started to exceed 10% in both regions in 2011 (Park, 2011). Thus, the decision to include data from 2011 provides a rationale for the sufficiency of data volume and the representativeness of each region. Furthermore, Google is one of the most popular search engines in both regions. Google's market share in the United States is 80%, which makes it the largest search engine, and 40% in South Korea, where it remains the second most popular search engine (StatCounter, 2025). Although Naver is the most frequently used search engine in South Korea, we selected Google as the target platform because Americans do not use Naver, and Google is the most common platform where users in both regions utilize their native languages to search for information. Due to this characteristic, English was utilized to search for information regarding health-related activities in the United States, whereas Korean was used for South Korea. Lastly, we decided to limit our data to December 2019 due to the explosive increase in searches for health-related keywords during the COVID 19 pandemic era (Stockwell et al., 2021). To rule out the temporary effects of COVID pandemic on individuals' search patterns, we focus on the period from 2011 to 2019.

### 3.3 Data analysis

To test H1 and the RQ, we used regression analyses. This study examines search volumes for “diet” and “exercise” across time periods and regions focusing on the early stages of the week (Monday), the month (1st day), and the year (January). In particular, this study treated the time period as a continuous variable (1 = Monday, 2 = Tuesday, to 7 = Sunday for the start of the week, 1 to 31 for the start of the month, and 1 = January, 2 = February to 12 = December for the start of the year), following guidelines from existing research (Dai et al., 2014). The search volumes for the target health-related terms, including “diet,” and “exercise,” and the placebo terms, including “news,” “weather,” and “YouTube” were then regressed on the time period, analyzed separately for both the United States and South Korea. Moreover, we downloaded and gathered the data focusing on 3-month periods and repeated this procedure several times until we obtained the complete dataset, as Google Trends only allows users to analyze search patterns over 3-month intervals (Dai et al., 2014). This characteristic of Google Trends led us to conduct regression analyses using fixed effects. Furthermore, this study conducted Augmented Dickey-Fuller (ADF) tests to analyze the stationarity of the data. All keyword search volume series, including both target and placebo terms, show appropriate stationarity, with ADF values ranging from −4.13 to −9.08, and *p*-values below 0.1. Furthermore, because the data has a time-series structure, regression models using Newey-West standard errors were checked to address potential heteroskedasticity and autocorrelation (Newey and West, 1987).

## 4 Results

### 4.1 Test of H1 and RQ

To test H1, we first analyzed Google Trends data for the United States. For the keyword “diet,” the regression results indicated that the start of the week negatively affected search volumes in the United States (*b* = −1.50, SE = 0.06, *p* < 0.001). Similarly, the start of the month (*b* = −0.13, SE = 0.03, *p* < 0.001) and year (*b* = −3.85, SE = 0.26, *p* < 0.001) also had negative effects on search volumes for “diet” in the United States. For the keyword “exercise,” the start of a weekly (*b* = −2.53, SE = 0.06, *p* < 0.001) and monthly (*b* = −0.10, SE = 0.02, *p* < 0.001) temporal cycles negatively affected search volumes in the United States. Furthermore, the start of the year had a negative effect on search volumes for “exercise” in the United States (*b* = −2.15, SE = 0.21, *p* < 0.001). In particular, the negative coefficient values indicate the pattern of fresh start phenomena, as the start of the temporal landmark was coded with the lowest value while the farthest point was coded with the highest. In other words, the volume of search patterns is highest at the initiation of the temporal landmark. Thus, the results collectively suggest that H1 was supported.

Focusing on Google Trends data in South Korea, the results indicated that the start of the week (*b* = −0.62, SE = 0.09, *p* < 0.001) and the year (*b* = −2.46, SE = 0.40, *p* < 0.001) negatively affected search volumes for “diet.” However, the effect associated with the start of the month was not significant (*b* = −0.02, SE = 0.03, *p* = 0.57). For the keyword of “exercise,” the start of the weekly temporal cycle (*b* = −1.24, SE = 0.07, *p* < 0.001) negatively influenced search volumes. Meanwhile, the start of the monthly (*b* = −0.04, SE =0.03, *p* = 0.11) and yearly (*b* = −0.36, *SE* = 0.33, *p* = 0.27) temporal cycles did not significantly impact search volumes for “exercise.” Collectively, these results indicate that Americans show a stronger fresh start phenomenon in searches for health-related activities, whereas such effects often disappear for Koreans. Table 1 displays the detailed results of the analysis.

**Table 1:** Regression analysis of Google search volume for diet and exercise.

Google search term	The United States		Korea	
	Diet	Exercise	Diet	Exercise
Start of the year (January)	−3.85^***^ (0.26)	−2.15^***^ (0.21)	−2.46^***^ (0.40)	−0.36 (0.33)
Start of the month (1st day)	−0.13^***^ (0.03)	−0.10^***^ (0.02)	−0.02 (0.03)	−0.04 (0.03)
Start of the week (Monday)	−1.50^***^ (0.06)	−2.53^***^ (0.06)	−0.62^***^ (0.09)	−1.24^***^ (0.07)
Fixed effects for each 3-month download	Yes	Yes	Yes	Yes
*R* ^2^	0.58	0.77	0.67	0.72

^***^p <0.001.

### 4.2 Test of placebo effects

This study tested whether the fresh start phenomenon also occur for placebo keywords in both societies. In the United States, the start of the month (*b* = −0.02, *SE* = 0.02, *p* = 0.27) was not significant for search volumes of the keyword “news.” However, unexpectedly, the weekly (*b* = −1.56, SE = 0.06, *p* < 0.001) and yearly (*b* = −0.74, SE = 0.21, *p* < 0.001) temporal cycles were significantly negative. In South Korea, the monthly (*b* = 0.03, SE = 0.02, *p* = 0.25) and yearly (*b* = 0.38, SE = 0.31, *p* = 0.22) cycles did not significantly affect the search volumes for “news,” while the weekly temporal cycle negatively impacted search volumes (*b* = −1.01, SE = 0.07, *p* < 0.001). Although the weekly and yearly cycles effects were significant for the search pattern of “news” which is similar to the pattern of the fresh start phenomenon, Dai et al. (2014) assert that such negative coefficient values are primarily caused by the decrease in the search pattern of “news” during the weekend.

For the keyword “weather,” the monthly (*b* = 0.01, SE = 0.04, *p* = 0.84) and yearly (*b* = −0.80, SE = 0.46, *p* = 0.08) temporal cycles did not significantly affect the search volumes in the United States. Although the start of the week (*b* = 0.48, SE = 0.11, *p* < 0.001) had a significant impact, the results showed the opposite pattern with a positive coefficient. In South Korea, the start of the month (*b* = 0.01, *SE* = 0.06, *p* = 0.91) and the start of the year (*b* = −1.29, SE = 0.71, *p* = 0.07) did not significantly affect the search volumes for “weather,” while the start of the week (*b* = 0.58, SE = 0.14, *p* < 0.001) had a positive effect on search volumes.

For the keyword “YouTube,” the start of the week (*b* = 2.44, SE = 0.08, *p* < 0.001) and the start of the month (*b* = 0.06, SE = 0.01, *p* < 0.001) positively impacted search volumes, while the start of the year (*b* = 0.06, SE = 0.15, *p* = 0.68) did not significantly affect search volumes in the United States. Similarly, the initiation of the week (*b* = 2.80, SE = 0.06, *p* < 0.001) and the year (*b* = 1.06, SE = 0.23, *p* < 0.001) had a positive effect on search volumes for “YouTube,” while the start of the month did not significantly affect search volumes (*b* =0.02, SE = 0.02, *p* = 0.25) in South Korea. The results collectively suggest that the fresh start phenomenon was not found for the terms “weather” and “YouTube” in either the United States or South Korea. Table 2 displays the detailed results for the placebo keywords.

**Table 2:** Regression analysis of Google search volume for news, weather, and YouTube.

Google search term	The United States			Korea		
	News	Weather	YouTube	News	Weather	YouTube
Start of the year (January)	−0.74^***^ (0.21)	−0.80 (0.46)	0.06 (0.15)	0.38 (0.31)	−1.29 (0.71)	1.06^***^ (0.23)
Start of the month (1st day)	−0.02 (0.02)	0.01 (0.04)	0.06^***^ (0.01)	0.03 (0.02)	0.01 (0.06)	0.02 (0.02)
Start of the week (Monday)	−1.56^***^ (0.06)	0.48^***^ (0.11)	2.44^***^ (0.08)	−1.01^***^ (0.07)	0.58^***^ (0.14)	2.80^***^ (0.06)
Fixed effects for each 3-month download	Yes	Yes	Yes	Yes	Yes	Yes
*R* ^2^	0.81	0.46	0.72	0.77	0.13	0.62

^***^p <0.001.

### 4.3 Additional analysis

**Figure 1:**
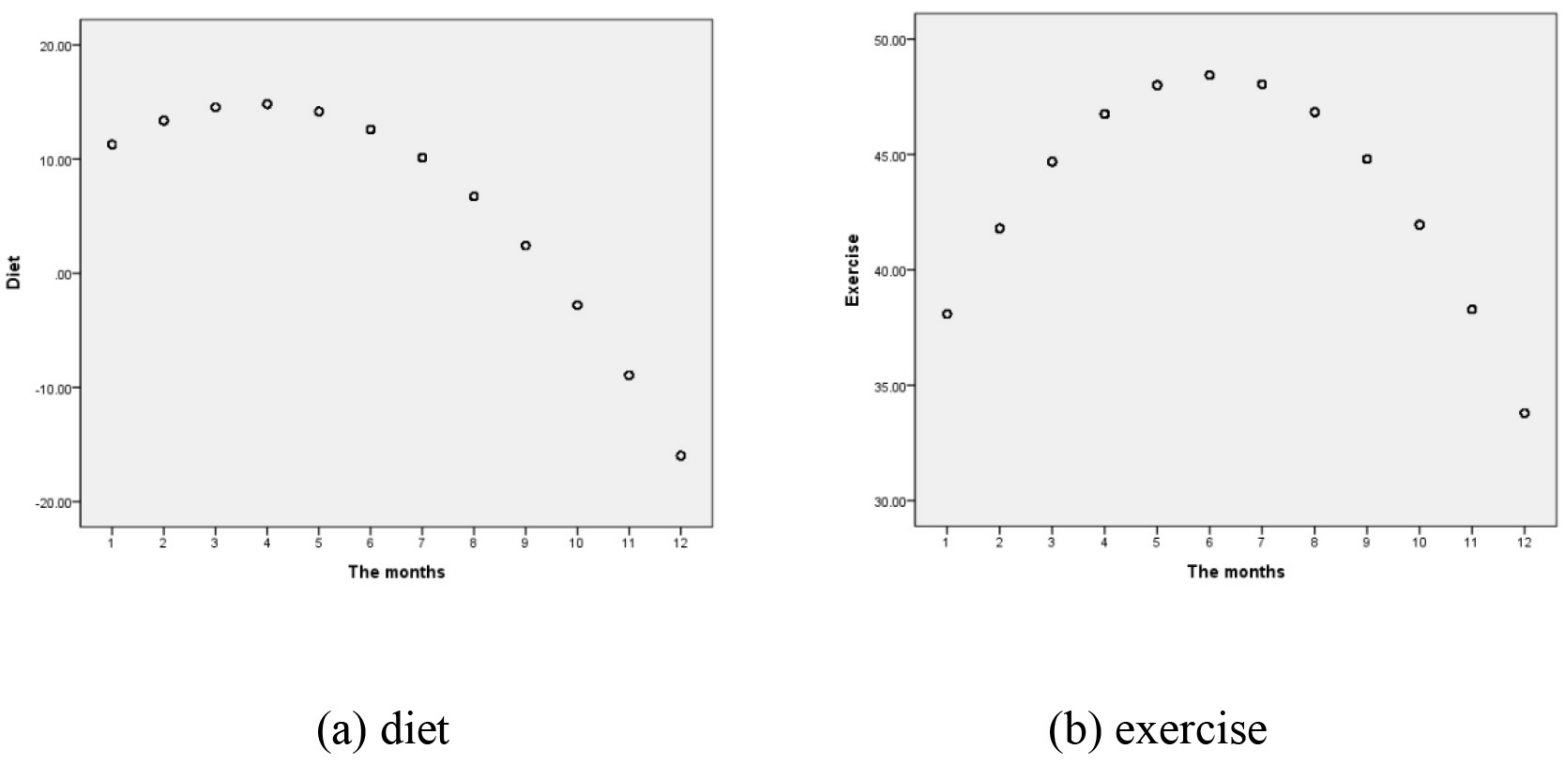
The estimated relationship between the months and Google search volume for diet and exercise terms in South Korea. **(a)** Diet. **(b)** Exercise.

We tested whether the search patterns for health-related terms exhibit an inverted U-shape in South Korea, which may be caused by the high degree of social anxiety during the summer. If social anxiety drives the search for health-related terms, search frequency should peak during the summer in collectivistic regions, but such a pattern is less likely to show in individualistic regions, as social anxiety due to external pressures is more prevalent among Koreans. In particular, we created a squared term for the year, but not for the week (Wednesday) or month (the 15th day of the month), as social anxiety regarding weight loss is unlikely to exist during such time periods. That is, people do not perceive stronger social pressure at the weekly or monthly levels, as the pressure for weight loss would remain consistent across all weeks and months. To test the inverted U-shape, we included both the linear and squared terms of the month in the regression. The results indicated that for the keyword “diet,” the coefficient for the month was significantly positive (*b* = 3.46, SE = 0.50, *p* < 0.001), while the coefficient for the squared month was negative and significant (*b* = −0.46, SE = 0.03, *p* < 0.001), which support an inverted U-shape relationship. In addition, the estimated turning point was approximately 3.79 months, which indicates the peak. Similarly, the coefficient for the month was significantly positive (*b* = 4.94, SE = 0.43, *p* < 0.001) for search volumes of “exercise” in South Korea, whereas the squared month was negatively significant (*b* = −0.41, SE = 0.03, *p* < 0.001), with an estimated turning point of approximately 6.02 months. In contrast, the inverted U-shaped search pattern for health-related keywords was not found in the United States. Particularly, the findings indicated that the coefficient for month was negative and significant (*b* = −4.15, SE = 0.38, *p* < 0.001) for the search volumes of “diet,” while the coefficient for the squared month was not significant (*b* = 0.02, SE = 0.03, *p* = 0.42). Similarly, the coefficient for the month was negative and significant (*b* = −2.45, SE = 0.39, *p* < 0.001) for the search volumes of “exercise,” whereas the coefficient for the squared month was not significant (*b* = 0.02, SE = 0.03, *p* = 0.50). The detailed results are presented in Table 3. The theoretical prediction curves based on the estimated coefficients for South Korea are illustrated in Figure 1.

**Table 3:** The inverted U-shaped relationship between the months and Google search volume for diet and exercise terms.

Google search term	The United States		Korea	
	Diet	Exercise	Diet	Exercise
The months	−4.15^***^ (0.38)	−2.45^***^ (0.39)	3.46^***^ (0.50)	4.94^***^ (0.43)
The months^2^	0.02 (0.03)	0.02 (0.03)	−0.46^***^ (0.03)	−0.41^***^ (0.03)
Turning point	–	–	3.79	6.02
Fixed effects for each 3-month download	Yes	Yes	Yes	Yes
*R* ^2^	0.49	0.63	0.69	0.72

^***^p <0.001.

## 5 Discussion

In this existing literature, scholars have examined how information search patterns on Google Trends are affected by the FSM (Dai et al., 2014). Thus, our study attempts to extend the relevant literature by focusing on how individuals' search patterns about health-related terms are not only impacted by FSM but also influenced by cultural contexts (individualistic vs. collectivistic cultures).

In general, our findings indicate that the search patterns differ between Americans and Koreans. In particular, the early stages of the week, month, and year were negatively associated with searches for “diet” and “exercise” in the United States. This pattern is consistent with the general notion of fresh start phenomenon. Past studies have found that the signal of a new start at a temporal landmark helps individuals mentally pay less attention to past failures and encourages them to set new goals to become a superior version of themselves (Yu et al., 2023). Indeed, adopting a big picture mindset driven by the emphasis on temporal landmarks creates the fresh start phenomenon, as such a mindset motivates individuals to imagine a positive image of themselves (Dai et al., 2014; Hennecke and Converse, 2017). Consistent with existing findings, Americans are more strongly motivated to search for health information on Google Trends when they perceive a specific calendar point as a new beginning (Dai et al., 2014).

Moreover, our findings also offer meaningful insights from a cultural perspective. People in individualistic cultures consider personal goals and achievements to be more important sociological values than collective goals (Oyserman et al., 2002). In this regard, aspirational behaviors related to individual health are often activated at the early stages of a time period for Americans (Dai et al., 2014). Therefore, we particularly examine cultural differences in the fresh start phenomenon by analyzing search patterns within a collectivistic society. The results indicate that the initiation of the week and the month (i.e., Monday and the 1st day of the month) created a fresh start effect for searches of “diet,” whereas this pattern was not observed during the first month of the year (i.e., January). Similarly, the early stages of the week and the month negatively predicted searches for “exercise,” which indicates the fresh start phenomenon, whereas the initiation of the year did not significantly predict searches for “exercise” in South Korea. Our findings collectively indicate that the fresh start phenomenon in the information search patterns for health-related terms is less evident among Koreans.

Although Americans and Koreans show distinct patterns of the fresh start phenomenon, our findings offer important theoretical implications from a cross-cultural perspective. Individuals' motivation and goal pursuits are influenced by culture (King and McInerney, 2014; Xu et al., 2023). Cross-cultural scholars have found that individuals who grow up in collectivistic regions generally experience stronger social anxiety compared to those in individualistic regions (Heinrichs et al., 2006). Thus, Koreans may experience heightened pressure to lose weight due to increased social anxiety during the summer period. In particular, the results of the quadratic regression model demonstrate an inverted U-shape for the South Korean data, whereas such a pattern was not detected in the United States. That said, Koreans show a stronger tendency to search for health-related terms on Google Trends, especially during the late spring and early summer (April to June) when social anxiety regarding weight loss emerges due to the thinner clothing styles compared to the fall and winter periods. Although our research does not directly test social anxiety effects, evidence from existing literature provides preliminary support for our argument. For example, Sun et al. (2009) found that individualism, along with uncertainty avoidance, negatively impacts public self-consciousness, which ultimately decreases individuals' healthy eating intention. Furthermore, Levine et al. (2016) demonstrated that interdependent self-construal, which is highly valued in collectivistic societies, has a positive impact on healthy eating behavior among Japanese individuals through a sense of positive relationship with others, whereas independent self-construal leads to healthy eating behavior among Americans through a sense of autonomy.

It is essential to mention that a few alternative explanations should be discussed before reaching a conclusion. Temporal landmarks may differ between collectivistic and individualistic cultures, as the Asian regions often follow lunar calendar events (Chen et al., 2025). For example, Koreans may perceive the New Year (Seollal) differently compared to Americans, who follow the Gregorian calendar. Thus, cultural differences in temporal landmarks may result in unintended confounding effects. Furthermore, there are some global events (e.g., Meatless Monday) that may possibly affect our findings. For example, Shakory et al. (2025) showed that meat consumption decreased significantly more for those who participated in the Meatless Monday campaign than for those who did not, even on days other than Monday. As Monday may perform as a temporal landmark similar to the fresh start phenomenon, such global events may also confound our findings. Thus, researchers should carefully interpret our findings or use more advanced methodological and data analysis approaches to rule out these alternative explanations.

Collectively, our findings provide unique theoretical and practical contributions from a cultural perspective. Particularly, the present study adds important knowledge to existing literature by investigating how the emphasis on temporal landmarks affects individuals' search patterns on Google Trends. Most importantly, as we are aware of, this study offers the first empirical evidence that shows the patterns of the fresh start phenomenon from a cross-cultural perspective by comparing search patterns on Google Trends between Americans and Koreans. In particular, as societies are structured around different cultural rules and norms (Han and Shavitt, 1994), this study demonstrates that people in individualistic cultures are more sensitive to temporal landmarks, whereas those in collectivistic cultures are less influenced by temporal landmarks and are instead affected more by societal circumstances, such as social pressure and anxiety.

Furthermore, our findings offer valuable insights for marketers and policymakers. Many organizations and government agencies run campaigns to encourage consumers to develop positive attitudes toward health-related activities or to promote their health-related initiatives. Recruiting consumers to actively engage in these campaigns is key to the success of health-related initiatives. Thus, launching health-related campaigns at the early stages of a time period, rather than at other time points may motivate consumers to engage more actively in the campaign, which would ultimately contribute to overall campaign success, especially for organizations operating in individualistic cultures. This strategy can also be applied to government agencies involved in public health. When such agencies aim to motivate the public to adopt healthier lifestyles or to disseminate health-related messages, launching campaigns at the early stages of a temporal period (e.g., Mondays) could effectively create fresh start phenomenon that promotes healthy eating and exercise. Moreover, in addition to utilizing certain time points as effective tactics, emphasizing a FSM in campaigns can further enhance the influence of temporal landmarks as messages designed to evoke a particular mindset have the power to affect individuals' attitudes and behaviors.

Similar to other academic works, this study also has some limitations that should be considered by other scholars. First, the datasets are limited to Google Trends within a specific time period. We made this decision because Google is a search engine that maintains high popularity in both regions and to minimize the influence of situational confounding effects (e.g., COVID 19). However, this methodological approach may also reduce the validity of our findings. For example, Google users in the United States may represent the general population, while users in South Korea may skew more toward the younger population. Although Google was the only choice to test cultural differences, as the target search engine should be used in both regions, researchers should try to test users' search patterns related to health-related activities by considering demographic characteristics as another key variable, as well as extending the time period. Researchers can also utilize cross-cultural experiments with a broader population. The use of experiments will also help researchers identify the key underlying mechanisms that drive the search patterns for both individualistic and collectivistic cultures. In fact, the current study did not focus on providing direct processing evidence (e.g., mediator) for social anxiety effects in collectivistic cultures. Furthermore, researchers can utilize observational studies or field experiments to test whether the fresh start mindset is strong enough to not only enhance individuals' interests but also change their actual behaviors. Second, similar to existing literature (e.g., Dai et al., 2014; Koo et al., 2020), the search terms are limited to “diet” and “exercise” in our analysis, which may reduce the external validity of the findings. Therefore, future scholars should expand the data by including more specific terms, such as “running,” or “gym,” to more effectively capture users' interests in health-related activities that are difficult to capture using a broader term. Lastly, our results may have been influenced by unintended confounding variables, such as situational circumstances (e.g., the fitness industry cycle, the Meatless Monday campaign, or lunar calendar dates), which could lead to alternative explanations. Although we utilized ADF tests and Newey-West standard errors and included placebo keywords to enhance the validity of our findings, researchers should carefully interpret our results or utilize more advanced data analytic approaches in future research.

## 6 Conclusions

Based on the data from Google Trends, this study examined whether the existence of temporal landmarks leads to the fresh start phenomenon across different cultural contexts. Given that Google Trends is an effective data management platform for capturing individuals' interests and search trends, and that the cross-cultural aspects of fresh start effects have been overlooked, the present study provides crucial implications in the fields of health marketing and policy management. We hope that scholars will use this study as a steppingstone to generate greater interest in the role of temporal landmarks.

## Data Availability

Publicly available datasets were analyzed in this study. This data can be found at: https://trends.google.co.kr/trends/.
